# P-1674. Point of Care (POC) Molecular Diagnostics for Pharyngitis in the Urgent Care (UC): Distinct Detection Patterns in Adult and Pediatric Patients Utilizing a Point-of-Care Multiplex PCR Panel (BIOFIRE® SPOTFIRE® ST)

**DOI:** 10.1093/ofid/ofaf695.1848

**Published:** 2026-01-11

**Authors:** Alexander J Lepak, Brittany Lehrer, Shari Barlow, Maureen Goss, Caroline Hamer, Cecelia He, Emily Temte, Sarah Walters, Jonathan Temte

**Affiliations:** University of Wisconsin School of Medicine and Public Health, Madison, WI; University of Wisconsin School of Medicine and Public Health, Madison, WI; University of Wisconsin School of Medicine and Public Health, Madison, WI; University of Wisconsin School of Medicine and Public Health, Madison, WI; University of Wisconsin School of Medicine and Public Health, Madison, WI; University of Wisconsin School of Medicine and Public Health, Madison, WI; University of Wisconsin School of Medicine and Public Health, Madison, WI; University of Wisconsin School of Medicine and Public Health, Madison, WI; University of Wisconsin School of Medicine and Public Health, Madison, WI

## Abstract

**Background:**

Pharyngitis is one of the most common medical conditions for UC visits and can be caused by a variety of viral or bacterial pathogens. Diagnostic uncertainty can lead to provider/patient frustration and inappropriate under-/over-prescribing of antimicrobials. SPOTFIRE ST (Sore Throat) is a novel, rapid, CLIA-waived POC testing platform for 14 common bacterial and viral pathogens; however, the detection characteristics of a multi-plex PCR test in a real-world UC setting has not been evaluated fully.

**Figure 1.**
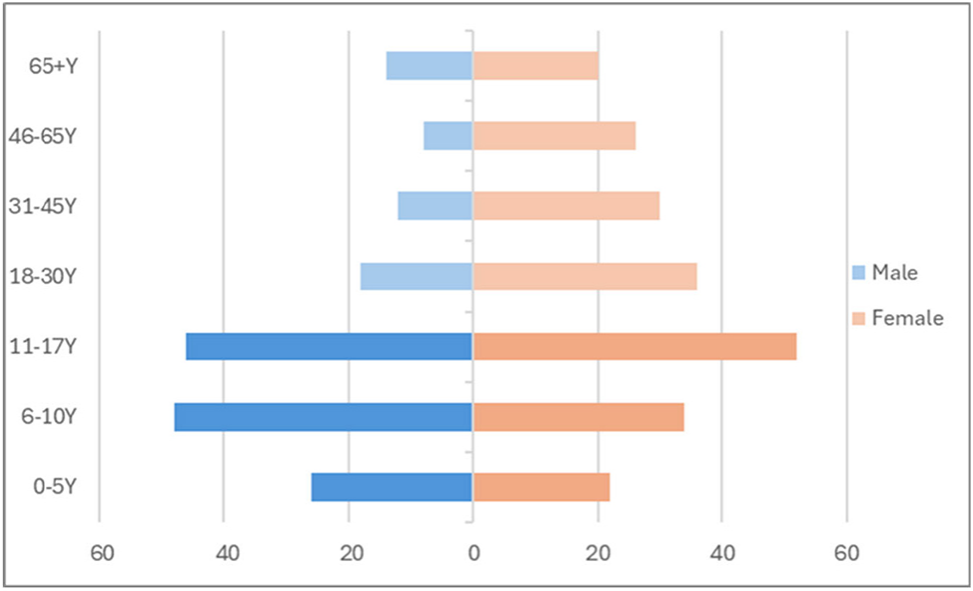
Age and Gender distribution of participants.

**Table 1.**
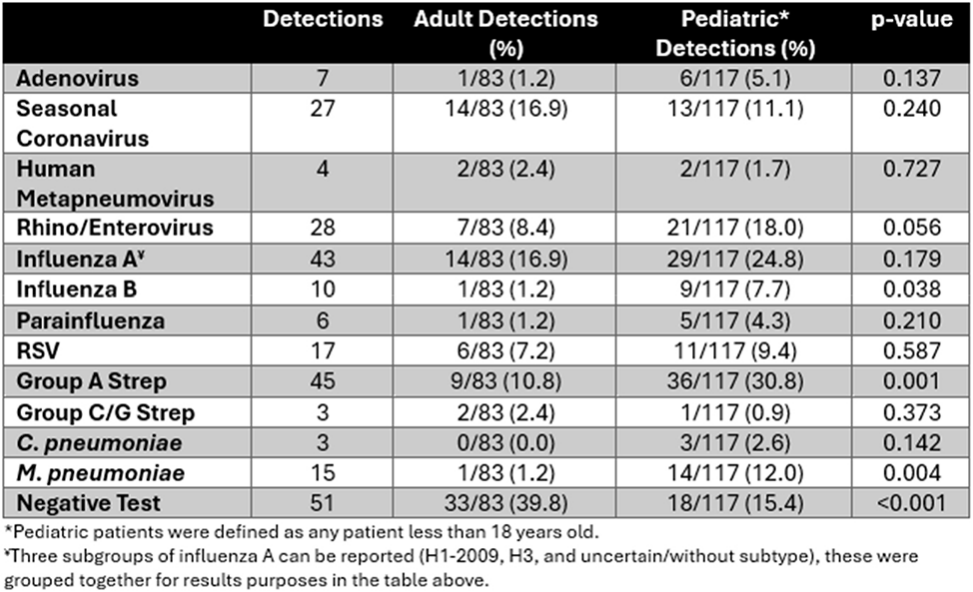
Pathogen Detection Results by Age Group.

**Methods:**

This prospective study was performed during peak respiratory illness season (Dec ‘24-Mar ‘25) at a single UC center (Madison, WI). Pediatric patients (PP) with fever (1-2 y) or pharyngitis (3-17 y) and adult patients (AP) with immunocompromising conditions or comorbid conditions with pharyngitis were eligible. After informed consent, research staff collected clinical information and an oropharyngeal swab from each participant. Specimens were tested using the SPOTFIRE ST machine according to instructions. Test results and survey data were recorded and analyzed using STATA (StataCorp, LLC).

**Table 2.**
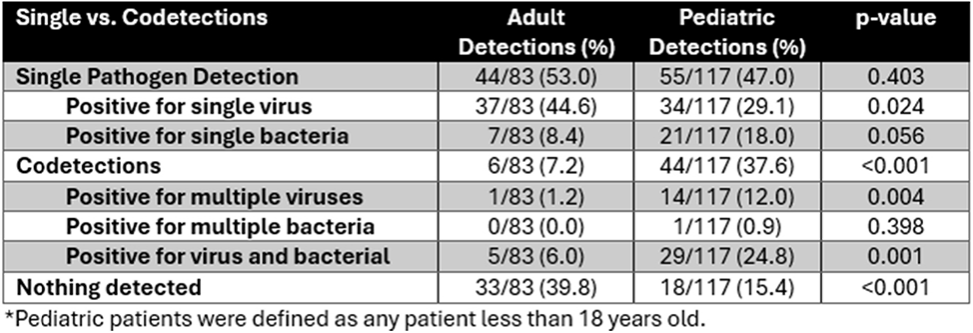
Single Detection and Codetections Among Adult and Pediatric Patients.

**Results:**

200 (117 PP, 83 AP, Fig 1) patients were enrolled, of which 149 (74.5%) had 208 total detections. PP had significantly higher detection rates (85% vs. 60%, p< 0.001). Table 1 lists pathogen results by age group. PP were statistically more likely to have Group A Streptococcus (GAS), influenza B, and *M. pneumoniae* infections. Multiple pathogen detection was common (n=50 patients). PP were significantly more likely to have codetections (38% vs 7%, p< 0.001), including multi-virus and bacteria-virus codetections (Table 2). Pathogens with specific antimicrobial treatments (influenza, GAS) were significantly more likely to be found in PP (68% vs. 28%, p< 0.001).

**Conclusion:**

SPOTFIRE ST demonstrated high detection rates in patients presenting to an UC with acute pharyngitis. PP had significantly higher detection rates, differences in pathogen detection (GAS, influenza B, and *M. pneumoniae*), higher rates of co-detections, and higher rates of pathogen detections treatable with specific antimicrobials. This test may be most useful in PP to guide specific antimicrobial therapy and in AP to prevent unnecessary therapy for viral diseases.

**Disclosures:**

Alexander J. Lepak, MD, FIDSA, BioMerieux: Grant/Research Support Jonathan Temte, MD, PhD, bioMérieux: Grant/Research Support

